# Photocatalytic CO_2_ Reduction Using Water as an Electron Donor under Visible Light Irradiation by Z-Scheme and Photoelectrochemical Systems over (CuGa)_0.5_ZnS_2_ in the Presence of Basic Additives

**DOI:** 10.1021/jacs.1c12636

**Published:** 2022-01-25

**Authors:** Shunya Yoshino, Akihide Iwase, Yuichi Yamaguchi, Tomiko M. Suzuki, Takeshi Morikawa, Akihiko Kudo

**Affiliations:** † Department of Applied Chemistry, Faculty of Science, Tokyo University of Science, 1-3 Kagurazaka, Shinjuku-ku, Tokyo 162-8601, Japan; ‡ Toyota Central R & D Laboratories., Inc., 41-1 Yokomichi, Nagakute, Aichi 480-1192, Japan

## Abstract

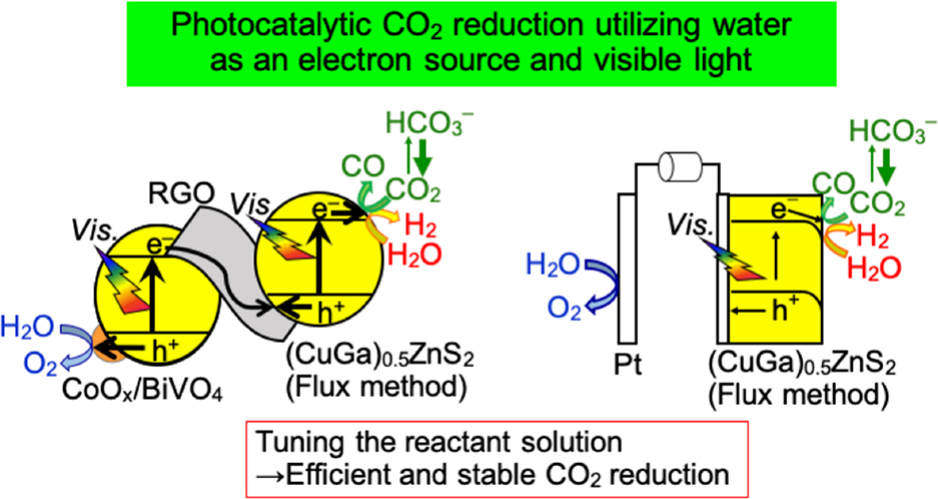

We demonstrated photocatalytic CO_2_ reduction using water as an electron donor under visible light irradiation by a Z-scheme photocatalyst and a photoelectrochemical cell using bare (CuGa)_0.5_ZnS_2_ prepared by a flux method as a CO_2_-reducing photocatalyst. The Z-scheme system employing the bare (CuGa)_0.5_ZnS_2_ photocatalyst and RGO-(CoO_*x*_/BiVO_4_) as an O_2_-evolving photocatalyst produced CO of a CO_2_ reduction product accompanied by H_2_ and O_2_ in a simple suspension system without any additives under visible light irradiation and 1 atm of CO_2_. When a basic salt (i.e., NaHCO_3_, NaOH, etc.) was added into the reactant solution (H_2_O + CO_2_), the CO formation rate and the CO selectivity increased. The same effect of the basic salt was observed for sacrificial CO_2_ reduction using SO_3_^2–^ as an electron donor over the bare (CuGa)_0.5_ZnS_2_ photocatalyst. The selectivity for the CO formation of the Z-schematic CO_2_ reduction reached 10–20% in the presence of the basic salt even in an aqueous solution and without loading any cocatalysts on the (CuGa)_0.5_ZnS_2_ metal sulfide photocatalyst. It is notable that CO was obtained accompanied by reasonable O_2_ evolution, indicating that water was an electron donor for the CO_2_ reduction. Moreover, the present Z-scheme system also showed activity for solar CO_2_ reduction using water as an electron donor. The bare (CuGa)_0.5_ZnS_2_ powder loaded on an FTO glass was also used as a photocathode for CO_2_ reduction under visible light irradiation. CO and H_2_ were obtained on the photocathode with 20% and 80% Faradaic efficiencies at 0.1 V vs RHE, respectively.

## Introduction

Beneficial CO_2_ fixation technology especially utilizing renewable energy is strongly demanded to solve resources, energy, and environmental issues. Photocatalytic CO_2_ reduction using water as an electron donor has been paid much attention as the promising reaction to convert solar energy to chemicals such as CO, HCOOH, and CH_4_, which is referred to as artificial photosynthesis.^1,2^ The photocatalytic CO_2_ reduction of an artificial photosynthesis has significant potential because chemical products such as CO are directly obtained from CO_2_ by utilizing sunlight and water, which is a chemically stable and abundant resource.^3^

**Figure 1:**
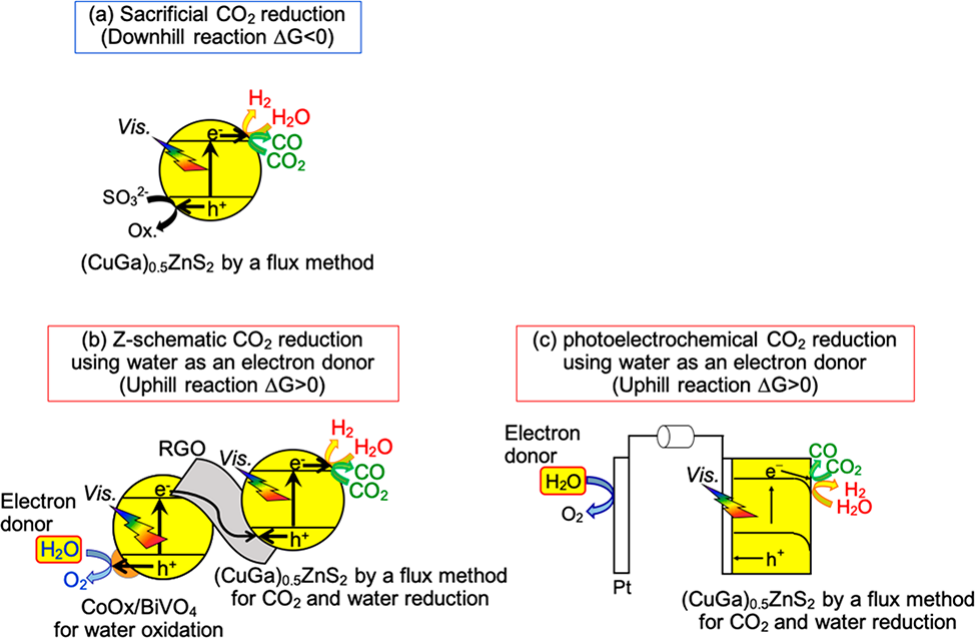
(a) CO_2_ reduction of a downhill reaction over a (CuGa)_0.5_ZnS_2_ photocatalyst using a sacrificial electron donor, (b) CO_2_ reduction of an uphill reaction by a Z-scheme photocatalyst system consisting of (CuGa)_0.5_ZnS_2_ and RGO-(CoO_*x*_/BiVO_4_), and (c) a photoelectrochemical system employing a (CuGa)_0.5_ZnS_2_ photocathode using water as an electron donor. An external bias lower than the redox potentials of CO_2_ and H_2_O should be applied for energy conversion in a photoelectrochemical cell.

The following points are key issues for photocatalytic CO_2_ reduction in artificial photosynthesis: high activity and selectivity, visible light response, using water as an electron donor, durability, and a simple aqueous suspension system. For achieving CO_2_ conversion in artificial photosynthesis, we must consider a Gibbs free energy change of the reaction (Δ*G*). Although photocatalytic CO_2_ reduction efficiently proceeds using sacrificial electron donors (i.e., triethanolamine and sulfite), the reaction is not artificial photosynthesis because of Δ*G* < 0, as shown in Figure 1(a). It is indispensable to reduce CO_2_ using water as an electron donor, namely, accompanied by O_2_ evolution by water oxidation, for achieving artificial photosynthesis with Δ*G* > 0. A Z-scheme photocatalyst and a photoelectrochemical cell are attractive systems to achieve CO_2_ reduction using water as an electron donor as shown in Figure 1(b) and (c).^4^ Moreover, these systems can widely employ visible light responsive photocatalysts aiming at efficient sunlight utilization. In a UV light responsive system using wide band gap photocatalysts, highly efficient photocatalytic CO_2_ reduction using water as an electron donor has been achieved in the last 10 years since the discovery of the Ag/BaLa_4_Ti_4_O_15_ photocatalyst via one-photon excitation.^5^ However, it cannot utilize visible and solar light because of its wide band gap. On the other hand, in a visible light responsive system, there are few suspension photocatalysts active for CO_2_ reduction using water as an electron donor accompanied by O_2_ evolution. So, construction of an efficient CO_2_ reduction system with a visible light response is demanded in the current stage. Ideally, the photocatalytic CO_2_ reduction system should be composed of powder-based photocatalyst materials for simplicity and low cost toward practical use.^6−9^ From the background, we have focused on metal sulfide powdered photocatalysts as a CO_2_-reducing photocatalyst in a Z-scheme system and a photocathode in a photoelectrochemical system.

Many metal sulfides are active for CO_2_ reduction under visible light irradiation in an aqueous solution containing a sacrificial reagent as an electron donor (Figure 1(a)).^10−13^ However, they cannot oxidize water to form O_2_ because photocorrosion (i.e., self-photooxidation) occurs in an aqueous solution in the absence of strong electron donors. In other words, the metal sulfides cannot reduce CO_2_ using water as an electron donor as a single-particulate photocatalyst. In contrast, we have successfully constructed a Z-schematic CO_2_ reduction system using water as an electron donor employing bare CuGaS_2_ as a CO_2_-reducing photocatalyst combined with CoO_*x*_/BiVO_4_ as an O_2_-evolving photocatalyst and reduced graphene oxide (RGO) as a solid-state electron mediator driven via interparticle electron transfer (Figure 1(b)).^12^ The Z-scheme photocatalyst system can reduce CO_2_ to CO accompanied by O_2_ evolution under visible light irradiation even using a photocorrosive metal sulfide photocatalyst.^12^ However, the CO evolution activity in the Z-schematic CO_2_ reduction was quite low and the CO selectivity was about 1%. Thus, improvement of the Z-schematic CO_2_ reduction system using metal sulfide and RGO-(CoO_*x*_/BiVO_4_) photocatalysts is a key issue for efficient CO_2_ fixation by an artificial photosynthesis.

Usage of a photocatalyst system that shows a high activity for water splitting under visible light irradiation is one approach to achieve efficient photocatalytic CO_2_ reduction. Recently, we have successfully improved a Z-schematic water splitting system using RGO-(CoO_*x*_/BiVO_4_) by combining it with Pt-loaded (CuGa)_0.5_ZnS_2_ prepared by a flux method as a H_2_-evolving photocatalyst instead of conventional Pt-loaded CuGaS_2_ prepared by a solid-state reaction (SSR).^14^ The Z-scheme photocatalyst system consisting of Pt/(CuGa)_0.5_ZnS_2_ by a flux method and RGO-(CoO_*x*_/BiVO_4_) shows the highest solar-to-hydrogen conversion efficiency among Z-schematic water splitting systems using a photocorrosive metal sulfide as a H_2_-evolving photocatalyst in a simple suspension system. It is expected that the (CuGa)_0.5_ZnS_2_ prepared by flux works for efficient Z-schematic CO_2_ reduction using water as an electron donor under visible light irradiation.

Photocatalytic CO_2_ reduction activity strongly depends on the condition of a reactant solution, especially pH and additives. For instance, the CO evolution rate and CO selectivity over cocatalyst-loaded wide band gap metal oxide photocatalysts are enhanced by adding a basic salt into a reactant solution because of pH adjustment and efficient supply of hydrated CO_2_ molecules of a reactant substrate.^15−17^ However, at the current stage, there are no reports on the effects of salt addition on Z-schematic CO_2_ reduction using metal sulfides as a reducing photocatalyst driven via interparticle electron transfer through RGO combined with an O_2_-evolving photocatalyst, except for usage of a [Ru(dpbpy)] complex as a CO_2_-reducing catalyst and a [Co(tpy)_2_] complex as an electron mediator.^13^ It is expected that the addition of a basic salt enhances the Z-schematic CO_2_ reduction employing metal sulfide and RGO-(CoO_*x*_/BiVO_4_) photocatalysts using water as an electron donor under visible light irradiation.

In addition to the Z-schematic CO_2_ reduction in a simple suspension system, a photoelectrochemical system also has potential to utilize a metal sulfide photocatalyst material for the CO_2_ reduction (Figure 1(c)). Although there are some reports on photoelectrochemical CO_2_ reduction using photocathodes of metal sulfides,^18−21^ it is still an important topic to develop a new photoelectrochemical CO_2_ reduction cell with a metal sulfide. (CuGa)_0.5_ZnS_2_ is a candidate as a new photocathode for the photoelectrochemical CO_2_ reduction because it has a p-type character and a suitable conduction band level for CO_2_ reduction.^13,14,22^

In the present study, we demonstrated Z-schematic CO_2_ reduction using bare (CuGa)_0.5_ZnS_2_ prepared by a flux method and RGO-(CoO_*x*_/BiVO_4_) using water as an electron donor under visible light irradiation in the presence of various salts as shown in Figure 1(b) as an artificial photosynthesis. Sacrificial CO_2_ reduction over bare (CuGa)_0.5_ZnS_2_, which is a half-reaction of Z-schematic CO_2_ reduction, was also investigated to support the data of the Z-scheme system (Figure 1(a)). We also conducted bulk electrolysis of photoelectrochemical CO_2_ reduction employing a bare (CuGa)_0.5_ZnS_2_ photocathode under visible light irradiation, as shown in Figure 1(c).

## Experimental Section

### Preparation of Photocatalysts

Powdered (CuGa)_0.5_ZnS_2_ was synthesized at 723 K for 15 h under vacuum by a flux method using a LiCl–CsCl flux (LiCl:CsCl = 3:2, melting point 600 K at the molar ratio of 3:2), according to a previous report.^14^ Starting materials of metal sulfides, Cu_2_S (Kojundo Chemical; 99%), Ga_2_S_3_ (Kojundo Chemical; 99.99%), and ZnS (Rare Metal Chemical; 99.99%), were mixed in an agate mortar with an atomic ratio of Cu:Ga:Zn = 1.0:1.2:2.4 for (CuGa)_0.5_ZnS_2_, containing 20 at. % of excess amounts of Ga and Zn. The obtained powders were washed with water to remove the flux reagent. Powdered (CuGa)_0.5_ZnS_2_ and CuGaS_2_ were also synthesized by an SSR method at 1073 and 873 K, respectively, for 10 h under vacuum according to previous reports as reference materials.^12,14^

BiVO_4_ was prepared by a liquid–solid-state reaction at room temperature, according to a previous report.^23^ A CoO_*x*_ cocatalyst was loaded on the BiVO_4_ by impregnation with an aqueous Co(NO_3_)_2_ (Wako; 99.5%) solution. The BiVO_4_ powder (0.5 g) and an aqueous Co(NO_3_)_2_ solution (80 mmol L^–1^, 0.53 mL) were placed in a porcelain crucible and dried on a boiling water bath. The Co(NO_3_)_2_-impregnated powder was calcined in air at 673 K for 2 h to obtain CoO_*x*_/BiVO_4_.^12^ An RGO-(CoO_*x*_/BiVO_4_) composite was prepared by photocatalytic reduction of a graphene oxide (NiSiNa materials; Rap TQ2-10) over CoO_*x*_/BiVO_4_ under visible light irradiation.^12,24^ CoO_*x*_/BiVO_4_ (0.3 g) and graphene oxide (5 wt % to CoO_*x*_/BiVO_4_) were dispersed in an aqueous methanol solution (50 vol %, 40 mL). The suspensions were stirred and bubbled with N_2_ gas under visible light irradiation for 3 h to prepare the RGO-(CoO_*x*_/BiVO_4_) composite.

(CuGa)_0.5_ZnS_2_ and RGO-(CoO_*x*_/BiVO_4_) powders were characterized before and after photocatalytic CO_2_ reduction. The crystal phases were clarified by X-ray diffraction (XRD; Rigaku; MiniFlex). Diffuse reflectance spectra were obtained using a UV–vis–NIR spectrometer (JASCO; Ubest-570) equipped with an integrating sphere and were transferred from reflection to absorbance by the Kubelka–Munk method. The surface component and chemical state were analyzed by X-ray photoelectron spectroscopy (XPS) and Auger electron spectroscopy (AES) using an X-ray photoelectron spectrometer (Shimazu; ESCA-3400) with a Mg Kα anode. The samples were loaded on a carbon tape or an indium foil for the XPS measurement. The chemical shift was corrected using a C 1s peak or an In 3d peak. Electron spin resonance (ESR) was measured at liquid nitrogen temperature (77 K) using an ESR spectrometer (ADANI; SPINSCAN) with a microwave frequency of 9.4 GHz.

### Photocatalytic Reactions

Sacrificial CO_2_ reduction (Figure 1(a)), which is a half-reaction of Z-schematic CO_2_ reduction, was conducted under 1 atm of CO_2_ gas flow (99.995%) at standard ambient temperature. Bare (CuGa)_0.5_ZnS_2_ photocatalyst (0.2 g) was dispersed in an aqueous solution containing 0.1 mol L^–1^ K_2_SO_3_ (Kanto Chemical; 95%) as a sacrificial reagent and 0–1.0 mol L^–1^ NaHCO_3_ (Wako Pure Chemical; 99.5%) in a top-irradiation reaction cell with a Pyrex window. Z-schematic water splitting and CO_2_ reduction using water as an electron donor (Figure 1(b)) were carried out at standard ambient temperature under 1 atm of Ar and CO_2_ gas flow, respectively. (CuGa)_0.5_ZnS_2_ and RGO-(CoO_*x*_/BiVO_4_) (0.05–0.1 g each) were dispersed in water (120 mL) in a top-irradiation cell with a Pyrex window. Li_2_CO_3_ (Kojundo Chemical; 99%), NaOH (Kanto Chemical; 95%), NaHCO_3_, Na_2_CO_3_ (Kanto Chemical; 99.8%), KHCO_3_ (Kanto Chemical; 99.5%), CsHCO_3_ (Wako Pure Chemical; 99%), NH_4_HCO_3_ (Kanto Chemical; 96.0%), H_3_BO_3_ (Kanto Chemical; 99.5%), and NaCl (Kanto Chemical; 99.5%) were added into the reactant solution, if necessary. A 300 W Xe lamp (PerkinElmer; CERMAX PE300BF) with a long-pass filter (HOYA; L42) was employed as a light source. The light irradiation area was 33 cm^2^. The power of incident light at the center was adjusted to 25 mW cm^–2^ at 520 nm using a band-pass filter (Asahi Spectra) and a photodiode head (OPHIR; PD300-UV head and NOVA display). A solar simulator (Asahi spectra; HAL-320; AM-1.5 G; 100 mW cm^–2^) was employed as a light source for photocatalytic solar CO_2_ reduction. The light irradiation area was 16 cm^2^. Amounts of evolved H_2_, O_2_, and CO were determined using an online gas chromatograph (Shimadzu; GC-8A, MS-5A column, TCD, Ar carrier for H_2_ and O_2_; Shimadzu; GC-8A, MS-13X column, FID with a methanizer, N_2_ carrier for CO). Formic acid of an aqueous product was analyzed using an ion chromatograph (IC; TOSOH; IC-2010, TSKgel SuperIC-Anion HR). The CO selectivity, the ratio of reacted electrons to holes, and the solar to chemical energy conversion efficiency were calculated as follows:

CO selectivity % = 100 × (rate of CO formation [μmol h^–1^])/(sum of rates of H_2_ and CO formations [μmol h^–1^])

e^–^/h^+^ = (2 × sum of rates of H_2_ and CO formations [μmol h^–1^])/(4 × rate of O_2_ formation [μmol h^–1^])

solar to chemical energy conversion efficiency % = 100 × ((Δ*G*°_298_ associated with water splitting × rate of H_2_ formation + Δ*G*°_298_ associated with CO_2_ reduction to CO using water as an electron donor × rate of CO formation [J mol^–1^ × μmol h^–1^])/(irradiation time [h] × solar energy (AM-1.5 G) [W cm^–2^] × irradiation area [cm^2^]))

### Photoelectrochemical Measurement

A (CuGa)_0.5_ZnS_2_ photocathode was prepared by a drop-cast method. Bare (CuGa)_0.5_ZnS_2_ powder (1 mg) was dispersed in 1 mL of ethanol (Kanto Chemical; 99.5%). The suspension was drop cast on a fluorine-doped tin oxide (FTO) transparent electrode. The (CuGa)_0.5_ZnS_2_-loaded FTO substrate was calcined at 573 K for 2 h under N_2_. Photoelectrochemical measurement was conducted using a three-electrode system with working, Ag/AgCl reference, and Pt counter electrodes connected to a potentiostat (Hokuto Denko; HSV-110) in an H-type glass cell with an optical window made of quartz. The glass cell was divided into cathode and anode parts using a Nafion membrane. An aqueous KHCO_3_ (Kanto Chemical; 99.5%) solution of an electrolyte was bubbled with CO_2_ gas at 15 mL min^–1^ during bulk electrolysis at −0.5 V vs Ag/AgCl at pH 6.9 (0.1 V vs RHE) (Figure 1(c)). A 300 W Xe lamp (PerkinElmer; CERMAX PE300BF) with a long-pass filter (HOYA; L42) was employed as a light source. The power of incident light at the center was adjusted to 25 mW cm^–2^ at 520 nm using a band-pass filter (Asahi Spectra) and a photodiode head (OPHIR; PD300-UV head and NOVA display). Amounts of evolved H_2_ and CO were determined using an online gas chromatograph (Shimadzu; GC-8A, MS-5A column, TCD, Ar carrier for H_2_; Shimadzu; GC-8A, MS-13X column, FID with a methanizer, N_2_ carrier for CO).

## Results and Discussion

### Z-Schematic CO_2_ Reduction under Visible Light Irradiation Using (CuGa)_0.5_ZnS_2_ and RGO-(CoO_*x*_/BiVO_4_) in the Presence of Various Salts in a Suspension System

Table 1 shows Z-schematic water splitting and CO_2_ reduction using bare (CuGa)_0.5_ZnS_2_ powder by a flux method as a reducing photocatalyst and RGO-(CoO_*x*_/BiVO_4_) as an O_2_-evolving photocatalyst under visible light irradiation. In a previous report, a Pt cocatalyst was loaded on the (CuGa)_0.5_ZnS_2_ for Z-schematic water splitting.^14^ However, the Pt cocatalyst does not work as an effective active site for CO_2_ reduction in Z-schematic CO_2_ reduction using a metal sulfide as a reducing photocatalyst,^11^ because Pt is well known to enhance water reduction and be poisoned by CO. So, first, Z-schematic water splitting was carried out dispersing (CuGa)_0.5_ZnS_2_ without any cocatalysts and RGO-(CoO_*x*_/BiVO_4_) in pure water under Ar and visible light irradiation (Table 1; entry 1). H_2_ and O_2_ steadily evolved in a stoichiometric ratio for 8 h under visible light irradiation (Figure S1). CO was not obtained, indicating RGO could be neglected as an origin of carbon-containing product. Thus, it was confirmed that the (CuGa)_0.5_ZnS_2_ worked as a H_2_-evolving photocatalyst even without cocatalyst in the Z-scheme photocatalyst system combined with RGO-(CoO_*x*_/BiVO_4_).

**Table 1:** Effect of Salt Addition on Z-Schematic CO_2_ Reduction under Visible Light Irradiation Using Bare (CuGa)_0.5_ZnS_2_ and RGO-(CoO_*x*_/BiVO_4_) Photocatalysts^a^

				activity [μmol h^–1^]				
entry	flow gas	additive [mmol L^–1^]	pH	H_2_	O_2_	CO	CO selectivity [%]	e^–^/h^+^
1	Ar	none	6.3	17.1	8.5	trace	0	1.0
2	CO_2_	none	4.1	1.1	0.5	0.1	11	1.23
3	Ar	H_2_SO_4_	4.0	1.4	0.7	trace	0	1.0
4	CO_2_	NaHCO_3_ (1)	5.2	3.5	1.9	0.4	11	1.04
5	CO_2_	NaHCO_3_ (10)	5.9	12.0	6.4	1.8	13	1.08
6	CO_2_	NaHCO_3_ (50)	6.7	8.3	3.8	2.4	23	1.20
7	CO_2_	NaHCO_3_ (100)	6.8	8.9	3.5	3.2	26	1.73
8	CO_2_	Li_2_CO_3_ (5)	5.9	8.1	4.4	2.3	22	1.17
9	CO_2_	NaOH (10)	5.8	10.2	5.2	1.4	12	1.07
10	CO_2_	Na_2_CO_3_ (10)	6.2	9.7	5.4	2.8	23	1.16
11	CO_2_	KHCO_3_ (1)	5.1	3.8	2.0	0.5	11	1.07
12	CO_2_	KHCO_3_ (10)	5.9	8.1	4.6	2.1	20	1.11
13	CO_2_	CsHCO_3_ (10)	5.9	8.3	4.3	2.4	23	1.24
14	CO_2_	NH_4_HCO_3_ (10)	5.9	9.4	5.0	2.4	20	1.17
15	CO_2_	H_3_BO_3_ (10)	4.1	1.2	trace	0.1	7	
16	CO_2_	NaCl (10)	4.1	0.4	trace	trace	0	

a Photocatalyst: 0.05 or 0.1 g each, reactant solution: water (120 mL), flow gas: CO_2_ and Ar (1 atm), light source: 300 W Xe lamp (λ > 420 nm), light irradiation area: 33 cm^2^, cell: top-irradiation cell with a Pyrex window. CO selectivity [%] = 100 × (rate of CO formation)/(sum of rates of H_2_ and CO formations).

Z-schematic CO_2_ reduction was carried out suspending the (CuGa)_0.5_ZnS_2_ and RGO-(CoO_*x*_/BiVO_4_) powders in CO_2_-dissolved water without any salts of additives under visible light irradiation. The pH of the reactant solution became around 4 because of dissolved CO_2_. CO of a CO_2_ reduction product was obtained in addition to H_2_ and O_2_ by water splitting (entry 2). We confirmed by IC analysis that HCOOH production was negligible in the Z-schematic CO_2_ reduction. We can rule out the possibility that obtained CO came from contaminants because CO was not obtained in a Z-schematic reaction under Ar even at pH 4 (entry 3). Thus, it was concluded that the Z-scheme photocatalyst system of bare (CuGa)_0.5_ZnS_2_ and RGO-(CoO_*x*_/BiVO_4_) reduced CO_2_ to CO using water as an electron donor under visible light irradiation.

The ability of the Z-scheme photocatalyst should depend on the pH of the reactant solution. An acidic condition resulted in low activity of the Z-scheme photocatalyst (entries 1–3). Thus, tuning the pH and salt addition are key issues for enhancing the Z-schematic CO_2_ reduction. The Z-schematic CO_2_ reduction was investigated at different pHs from 4 to 7 adjusted with various concentrations of NaHCO_3_ of a basic additive (entries 2, 4–7). CO, H_2_, and O_2_ evolved at any pH under visible light irradiation, but negligible HCOOH was obtained (<0.1 μmol h^–1^). Adjusting the pH to 5–6 by addition of 1–10 mmol L^–1^ NaHCO_3_ (entries 4, 5) enhanced H_2_, O_2_, and CO formation compared with pH 4 without any additives (entry 2). The ratio of reacted electrons to holes (e^–^/h^+^) was estimated to be almost 1 in the Z-schematic CO_2_ reduction (entries 4, 5). However, when the concentration of NaHCO_3_ was equal to or higher than 50 mmol L^–1^, CO formation was more enhanced but O_2_ evolution was not. The increase in the selectivity for CO formation in the presence of a high concentration of NaHCO_3_ was due to an efficient supply of hydrated CO_2_ molecules of a reactant substrate. However, when 100 mmol L^–1^ of NaHCO_3_ was used, the e^–^/h^+^ was 1.73, being exceedingly beyond unity (entry 7). We confirmed that H_2_O_2_ of another candidate as a water oxidation product was not detected after the Z-schematic CO_2_ reduction. In the Z-schematic water splitting at around neutral pH (entry 1), stoichiometric amounts of H_2_ and O_2_ were obtained. In other words, e^–^/h^+^ being beyond unity at around neutral pH was observed in Z-schematic CO_2_ reduction but not Z-schematic water splitting. These results implied that photocorrosion of a metal sulfide photocatalyst might be accelerated in Z-schematic CO_2_ reduction in an aqueous solution containing 100 mmol L^–1^ NaHCO_3_.

**Figure 2:**
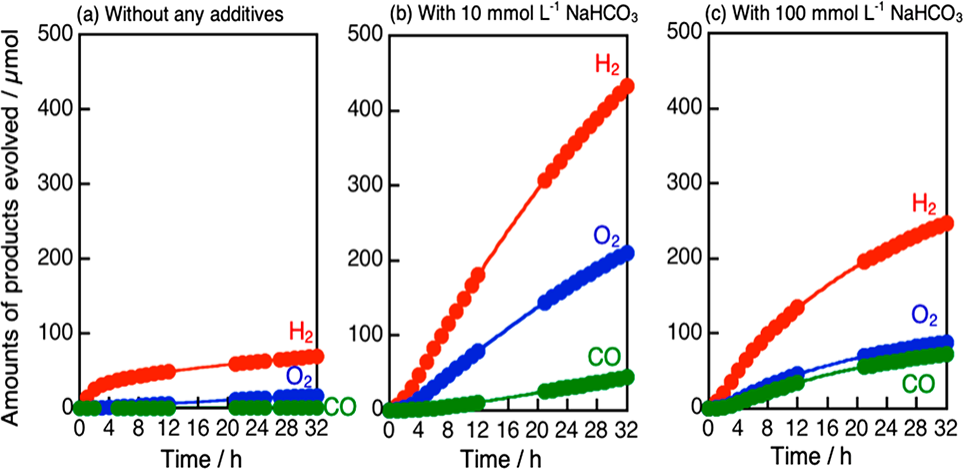
Z-schematic CO_2_ reduction under visible light irradiation using (CuGa)_0.5_ZnS_2_ and RGO-(CoO_*x*_/BiVO_4_) photocatalysts (a) without any additives, (b) with 10 mmol L^–1^ NaHCO_3_, and (c) with 100 mmol L^–1^ NaHCO_3_. Photocatalyst: 0.1 g each, reactant solution: 120 mL, flow gas: CO_2_ (1 atm), light source: 300 W Xe lamp (λ > 420 nm), light irradiation area: 33 cm^2^, cell: top-irradiation cell with a Pyrex window.

Figure 2 shows time courses of Z-schematic CO_2_ reduction using bare (CuGa)_0.5_ZnS_2_ and RGO-(CoO_*x*_/BiVO_4_) in the absence and the presence of 10 or 100 mmol L^–1^ NaHCO_3_, corresponding to Table 1, entries 2, 5, and 7. H_2_, O_2_, and CO continuously evolved for 32 h under visible light irradiation in all cases. The CO_2_ reduction activity was quite low at pH 4 in the absence of NaHCO_3_ (Figure 2(a)). In contrast, addition of NaHCO_3_ much enhanced the CO_2_ reduction (Figure 2(b), (c)). The unfavorable Z-scheme photocatalyst ability at pH 4 would be due to damage of the metal sulfide material because the pH might be too low for the metal sulfide employed in the present study. When NaHCO_3_ with a high concentration of 100 mmol L^–1^ was used, obvious deactivation was observed even under neutral pH, though CO formation was significantly enhanced (Figure 2(c)). The deactivation was suppressed at the low concentration of 10 mmol L^–1^ NaHCO_3_ (Figure 2(b)). The Z-scheme photocatalyst in 10 mmol L^–1^ NaHCO_3_ maintained 80% of the highest activity at 6–22 h. The turnover number of the photocatalytic reaction was calculated to see if the CO_2_ reduction proceeded photocatalytically. The turnover number of a molar quantity of reacted electrons used for the CO and H_2_ formations to that of the employed (CuGa)_0.5_ZnS_2_ was calculated to be 1.9 at 32 h, as shown in Figure 2(b). No peeled RGO was observed in the reactant solution after the Z-schematic CO_2_ reduction. In addition, the products were not obtained under dark conditions. These results indicated the Z-schematic CO_2_ reduction proceeded photocatalytically. Thus, a low concentration of NaHCO_3_ (10 mmol L^–1^) was suitable to enhance and stabilize the Z-schematic CO_2_ reduction using water as an electron donor over bare (CuGa)_0.5_ZnS_2_ and RGO-(CoO_*x*_/BiVO_4_) under visible light irradiation.

The effects of addition of acidic, neutral, and other basic salts in a reactant solution on the Z-schematic CO_2_ reduction were also investigated. The addition of basic salts of Li_2_CO_3_, NaOH, Na_2_CO_3_, KHCO_3_, CsHCO_3_, and NH_4_HCO_3_ enhanced Z-schematic CO_2_ reduction using water as an electron donor (Table 1; entries 8–14). HCOOH was not obtained with any basic additives, indicating CO was produced as a CO_2_ reduction product being independent of the kind of additives in this experimental condition. Entry 9 (with 10 mmol L^–1^ NaOH) is in the same line as the entry 5 (with 10 mmol L^–1^ NaHCO_3_) because the addition of alkaline compounds such as NaOH in the CO_2_ flowing system gives HCO_3_^–^, being similar to the condition of entry 5. Basic salts gave positive effects of “pH adjustment” and “efficient supply of hydrated CO_2_” for enhancement of the Z-schematic CO_2_ reduction. In contrast to the basic salts, the acidic salt H_3_BO_3_ and the neutral salt NaCl gave negative effects for not only the Z-schematic CO_2_ reduction but also the Z-schematic water splitting (entries 15, 16). H_3_BO_3_ and NaCl additions were not suitable for Z-schematic water splitting and CO_2_ reduction because the pH of the CO_2_-saturated aqueous solution was around 4. Moreover, adsorption of ions from H_3_BO_3_ and NaCl salts on a photocatalyst surface might change the surface and, therefore, suppress the contact between CO_2_-reducing and O_2_-evolving photocatalyst particles, resulting in a low-efficient interparticle electron transfer. We also confirmed K_2_SO_4_ salt addition gave a negative effect for Z-schematic water splitting even in the absence of CO_2_.

**Figure 3:**
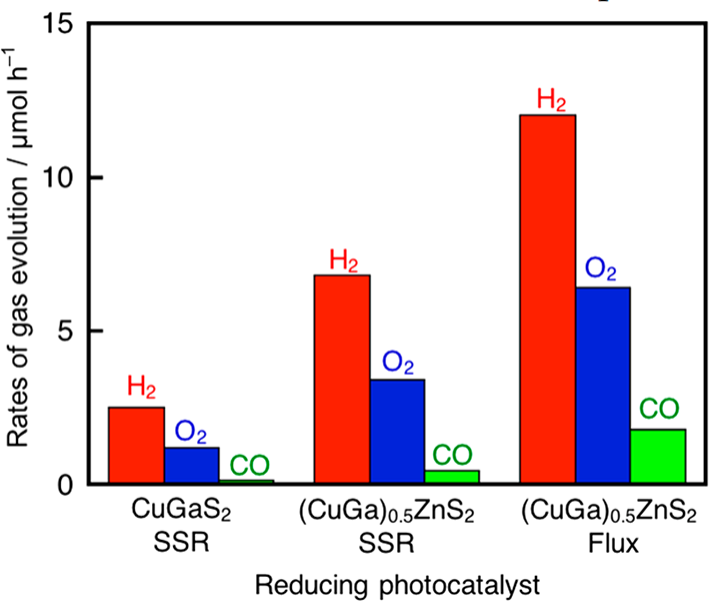
Z-schematic CO_2_ reduction under visible light irradiation using CuGaS_2_ or (CuGa)_0.5_ZnS_2_ as a reducing photocatalyst combined with an RGO-(CoO_*x*_/BiVO_4_) photocatalyst in the presence of 10 mmol L^–1^ NaHCO_3_. Photocatalyst: 0.05–0.1 g each, reactant solution: 10 mmol L^–1^ NaHCO_3_ (120 mL), flow gas: CO_2_ (1 atm), light source: 300 W Xe lamp (λ > 420 nm), light irradiation area: 33 cm^2^, cell: top-irradiation cell with a Pyrex window. CuGaS_2_ was prepared by an SSR at 873 K for 10 h, and (CuGa)_0.5_ZnS_2_ was prepared by an SSR at 1073 K for 10 h or by a flux at 723 K for 15 h.

To compare the ability for photocatalytic CO_2_ reduction between Z-scheme systems developed in the present and previous studies, we also examined the Z-schematic CO_2_ reduction using our previous Z-scheme system consisting of bare CuGaS_2_ by an SSR and RGO-(CoO_*x*_/BiVO_4_)^12^ in the presence of 10 mmol L^–1^ NaHCO_3_, which was the optimized concentration in the present study as summarized in Figure 3. Z-schematic CO_2_ reduction continuously proceeded even using CuGaS_2_ (Figure S2(a)). The selectivity for CO formation in the Z-schematic CO_2_ reduction using CuGaS_2_ with 10 mmol L^–1^ NaHCO_3_ reached 5.3%, which was higher than the previously reported selectivity of about 1% without any additives,^12^ indicating NaHCO_3_ addition was effective for Z-schematic CO_2_ reduction using not only (CuGa)_0.5_ZnS_2_ but also CuGaS_2_. Moreover, Z-schematic CO_2_ reduction using (CuGa)_0.5_ZnS_2_ prepared by an SSR not by a flux steadily proceeded in the presence of 10 mmol L^–1^ NaHCO_3_ under visible light irradiation (Figure S2(b)). The present Z-scheme system using bare (CuGa)_0.5_ZnS_2_ prepared by a flux as a reducing photocatalyst showed the highest CO formation rate and CO selectivity compared with CuGaS_2_ and (CuGa)_0.5_ZnS_2_ prepared by an SSR, as shown in Figure 3. Thus, we successfully improved Z-schematic CO_2_ reduction using water as an electron donor under visible light irradiation by applying (CuGa)_0.5_ZnS_2_ prepared by a flux method and adding a basic salt into the reactant solution. It is notable that the Z-scheme system can produce CO with a selectivity of 10–20% accompanied by almost stoichiometric O_2_ evolution under visible light irradiation in a simple suspension system in an aqueous medium even without special surface modification of a metal sulfide photocatalyst.

**Figure 4:**
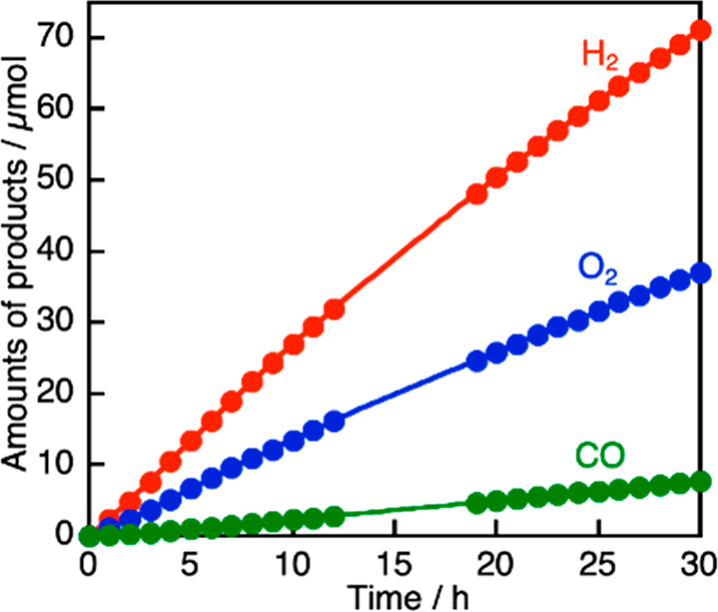
Z-schematic solar CO_2_ reduction using (CuGa)_0.5_ZnS_2_ prepared by a flux method and RGO-(CoO_*x*_/BiVO_4_) photocatalysts in the presence of 10 mmol L^–1^ NaHCO_3_. Photocatalyst: 0.1 g each, reactant solution: 10 mmol L^–1^ NaHCO_3_ (120 mL), flow gas: CO_2_ (1 atm), light source: simulated sunlight (AM-1.5 G), light irradiation area: 16 cm^2^, cell: top-irradiation cell with a Pyrex window.

We also carried out Z-schematic solar CO_2_ reduction using the improved Z-scheme system consisting of (CuGa)_0.5_ZnS_2_ prepared by a flux method and RGO-(CoO_*x*_/BiVO_4_). Z-schematic CO_2_ reduction proceeded under simulated sunlight with almost unity e^–^/h^+^ for 30 h, as shown in Figure 4. The solar to chemical energy conversion efficiency was 0.012%. Therefore, we successfully demonstrated artificial photosynthetic solar CO_2_ reduction in a simple powdered suspension system.

### Sacrificial CO_2_ Reduction under Visible Light Irradiation over (CuGa)_0.5_ZnS_2_ Photocatalyst in the Presence of NaHCO_3_ in a Suspension System

CO_2_ reduction and H_2_ evolution over (CuGa)_0.5_ZnS_2_ prepared by a flux method from an aqueous solution containing K_2_SO_3_ as a sacrificial electron donor were investigated at various pHs as a half-reaction of Z-schematic CO_2_ reduction as shown in Table 2. When sacrificial CO_2_ reduction was carried out using an aqueous solution with K_2_SO_3_ in the absence of NaHCO_3_ (entry 1), (CuGa)_0.5_ZnS_2_ produced CO in addition to H_2_ under visible light irradiation. The formation of HCOOH was negligible. Twenty-three percent of the high CO selectivity was obtained, even though any cocatalysts including a metal complex catalyst^13,25^ working as a CO_2_ reduction site were not loaded on the (CuGa)_0.5_ZnS_2_. As the concentration of NaHCO_3_ and pH became high, the CO evolution rate and CO selectivity became high (entries 1–4). The enhancement of CO selectivity was due to an efficient supply of hydrated CO_2_ molecules of a reactant species in the presence of NaHCO_3_ that worked as a CO_2_ buffer.^15,16^ On the other hand, the enhancement of H_2_ and CO evolution, namely, an increase in the number of reacted electrons, was caused by an increase in the concentration of SO_3_^2–^ ions working as a sacrificial electron donor at high pH. The ratio of SO_3_^2–^ to HSO_3_^–^ in an aqueous solution depends on the follow equation:
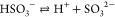
The p*K*_a_ of the equation is 6.91.^26^ Therefore, we also evaluated the pH dependence of sacrificial H_2_ evolution over bare (CuGa)_0.5_ZnS_2_ under 1 atm of Ar flow (entries 5–8). The H_2_ evolution rates, namely, the number of reacted electrons, at pH 7.6 and 9.7 (entries 7, 8) were larger than those at pH 6.6–6.9 (entries 5, 6). Thus, when SO_3_^2–^ mainly existed at 6.9 < pH, a high H_2_ evolution rate was obtained. On the other hand, the H_2_ evolution rate decreased due to the decrease in SO_3_^2–^ of a sacrificial electron donor at pH ≤ 6.9. A similar pH dependence of sacrificial H_2_ evolution activity has been reported over a bare ZnS photocatalyst using SO_3_^2–^ as an electron donor.^26^ In the sacrificial CO_2_ reduction over (CuGa)_0.5_ZnS_2_ under visible light irradiation, 1.0 mol L^–1^ NaHCO_3_ addition improved not only CO selectivity by efficient supply of hydrated CO_2_ molecules of a reactant species but also the number of reacted electrons because of the increase in SO_3_^2–^ as a sacrificial electron donor. Thus, NaHCO_3_ addition was also effective for enhancement of sacrificial CO_2_ reduction over bare (CuGa)_0.5_ZnS_2_ as well as Z-schematic CO_2_ reduction using (CuGa)_0.5_ZnS_2_ as a CO_2_-reducing photocatalyst. It is stressed that (CuGa)_0.5_ZnS_2_ has potential to reduce CO_2_ to CO with a selectivity of about 40% by only tuning a reactant solution even without any cocatalysts and special surface modification.

**Table 2:** Effect of pH on Sacrificial CO_2_ Reduction and H_2_ Evolution under Visible Light Irradiation over a Bare (CuGa)_0.5_ZnS_2_ Photocatalyst from an Aqueous Solution Containing K_2_SO_3_ as a Sacrificial Reagent^a^

				activity [μmol h^–1^]		
entry	flow gas	additive [mol L^–1^]	pH	H_2_	CO	CO selectivity [%]
1	CO_2_	none	6.6	6.5	1.9	23
2	CO_2_	NaHCO_3_ (0.1)	6.9	6.3	2.5	28
3	CO_2_	NaHCO_3_ (0.5)	7.2	9.2	5.0	35
4	CO_2_	NaHCO_3_ (1.0)	7.4	15.9	11.7	42
5	Ar	H_2_SO_4_	6.6	16.5		
6	Ar	H_2_SO_4_	6.9	16.2		
7	Ar	H_2_SO_4_	7.6	32.4		
8	Ar	none	9.7	35.8		

a Photocatalyst: 0.2 g, reactant solution: 0.1 mol L^–1^ K_2_SO_3_ aqueous solution (120 mL), flow gas: CO_2_ or Ar (1 atm), light source: 300 W Xe lamp (λ > 420 nm), light irradiation area: 33 cm^2^, cell: top-irradiation cell with a Pyrex window. CO selectivity [%] = (rate of CO formation)/(sum of rates of H_2_ and CO formation).

**Figure 5:**
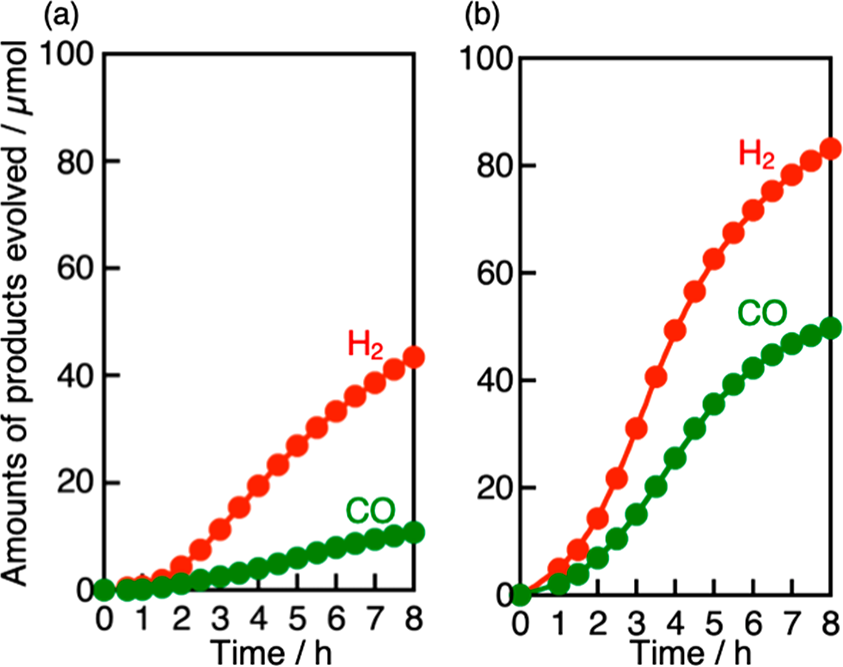
Sacrificial CO_2_ reduction under visible light irradiation over a (CuGa)_0.5_ZnS_2_ photocatalyst from an aqueous solution containing 0.1 mol L^–1^ K_2_SO_3_ as a sacrificial reagent (a) without NaHCO_3_ and (b) with 1.0 mol L^–1^ NaHCO_3_. Photocatalyst: 0.2 g, reactant solution: 120 mL, flow gas: CO_2_ (1 atm), light source: 300 W Xe lamp (λ > 420 nm), light irradiation area: 33 cm^2^, cell: top-irradiation cell with a Pyrex window.

Figure 5 shows the time courses of sacrificial CO_2_ reduction over (CuGa)_0.5_ZnS_2_ in the presence of 0.1 mol L^–1^ K_2_SO_3_ as a sacrificial electron donor with and without 1.0 mol L^–1^ NaHCO_3_ under visible light irradiation corresponding to Table 2, entries 1 and 4. CO was produced accompanied by H_2_ evolution. The turnover number of reacted electrons used for the CO and H_2_ formation to S atoms on the surface of (CuGa)_0.5_ZnS_2_ powders was estimated to be 15.5 at 8 h, by calculating the sum of S atoms on the surface calculated from the specific surface area^14^ and the (111) lattice plane in Figure 5(b). Moreover, H_2_ and CO were not obtained under dark conditions. These results indicated the sacrificial CO_2_ reduction over (CuGa)_0.5_ZnS_2_ proceeded photocatalytically. However, the CO_2_ reduction activity decreased at the later period. Similar behavior was also previously reported for a bare CuGaS_2_ photocatalyst.^11^ The deactivation was more significant with 1.0 mol L^–1^ NaHCO_3_ than without the additive. It is difficult to examine the sacrificial reaction with a concentration higher than 1 mol L^–1^ NaHCO_3_ because of the solubility. The deactivation in the sacrificial CO_2_ reduction (Figure 5) was more significant than that in the Z-schematic CO_2_ reduction system in the absence of a sacrificial electron donor (Figure 2(b)). In sacrificial CO_2_ reduction, SO_3_^2–^ should be photooxidized to SO_4_^2–^, which is an inert ion. If produced SO_4_^2–^ ions irreversibly adsorbed on a metal sulfide photocatalyst, it would suppress adsorption of SO_3_^2–^ as a reactant. The suppression of SO_3_^2–^ adsorption might lead to self-photooxidation of a metal sulfide photocatalyst. In addition, a localized pH gradient might damage the metal sulfide powder. In contrast to the sacrificial reaction, photogenerated holes on a metal sulfide photocatalyst are continuously consumed by photogenerated electrons on BiVO_4_ via interparticle electron transfer through RGO without negative effects of the ions, resulting in more steady CO_2_ reduction in a Z-scheme system.

### Characterization of Photocatalyst Powders after Z-Schematic and Sacrificial CO_2_ Reduction

Z-scheme photocatalysts after the CO_2_ reduction in the presence of 10 and 100 mmol L^–1^ NaHCO_3_ (Figure 2(b), (c)) and (CuGa)_0.5_ZnS_2_ after the sacrificial CO_2_ reduction with and without 1.0 mol L^–1^ NaHCO_3_ (Figure 5) were characterized by XRD, DRS, AES, and ESR to reveal the cause of deactivation. There was no significant difference in XRD patterns of (CuGa)_0.5_ZnS_2_ before and after the sacrificial CO_2_ reduction (Figure S3(a), (f), (g)). Moreover, XRD patterns of a mixture of (CuGa)_0.5_ZnS_2_ and RGO-(CoO_*x*_/BiVO_4_) of the Z-scheme photocatalysts did not change before and after Z-schematic CO_2_ reduction (Figure S3(c)–(e)). The above indicated that the crystal structure of (CuGa)_0.5_ZnS_2_ and BiVO_4_ did not change and no crystalline impurity phases existed even after the sacrificial CO_2_ reduction and the Z-schematic CO_2_ reduction.

**Figure 6:**
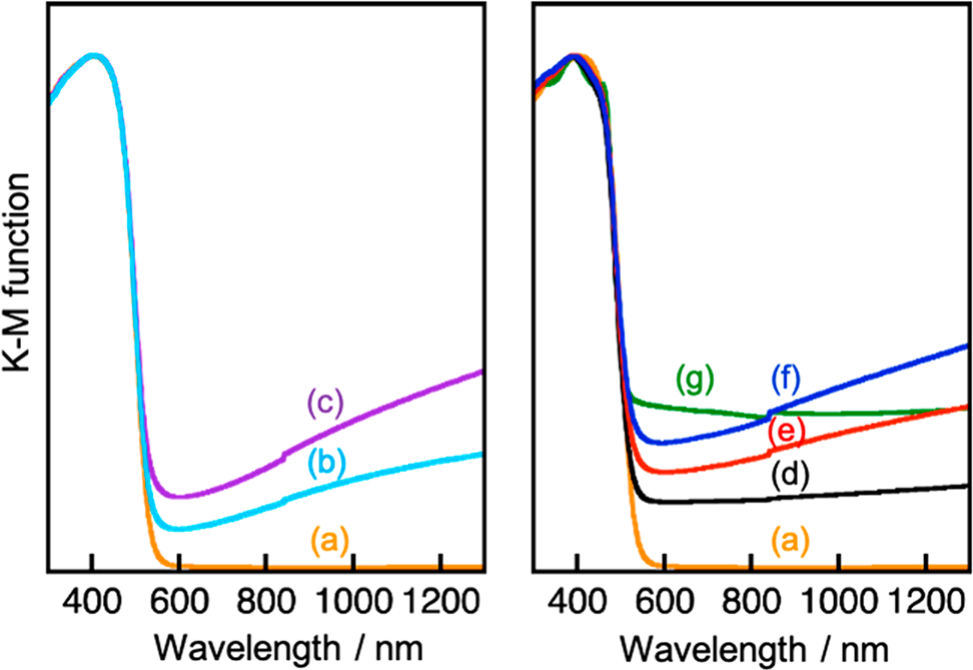
Diffuse reflectance spectra of (a) (CuGa)_0.5_ZnS_2_, (CuGa)_0.5_ZnS_2_ after sacrificial CO_2_ reduction (b) without NaHCO_3_ and (c) with 1.0 mol L^–1^ NaHCO_3_, a Z-scheme photocatalyst of a mixture of (CuGa)_0.5_ZnS_2_ and RGO-(CoO_*x*_/BiVO_4_) (d) before CO_2_ reduction and after CO_2_ reduction (e) using 10 mmol L^–1^ NaHCO_3_ and (f) using 100 mmol L^–1^ NaHCO_3_, and (g) RGO-(CoO_*x*_/BiVO_4_).

We also measured diffuse reflectance spectroscopy (DRS) of samples before and after CO_2_ reduction as shown in Figure 6. First, we see (CuGa)_0.5_ZnS_2_ before and after the sacrificial CO_2_ reduction. (CuGa)_0.5_ZnS_2_ before CO_2_ reduction had an obvious band edge without a risen baseline (Figure 6(a)). In contrast, (CuGa)_0.5_ZnS_2_ after the sacrificial CO_2_ reduction obviously possessed a risen baseline (Figure 6(b), (c)), especially using NaHCO_3_. XPS analysis indicated that the surface composition of Ga, Zn, and S to Cu decreased after sacrificial CO_2_ reduction (Table S1), suggesting the Ga, Zn, and S might dissolve into the reactant solution during the sacrificial CO_2_ reduction. These DRS and XPS measurements implied that some defect levels and/or Cu-containing impurities existed on the metal sulfide surface after the sacrificial CO_2_ reduction, resulting in absorbing light up to the near-IR region. Moreover, the alternation after the sacrificial CO_2_ reduction using NaHCO_3_ was more significant than that without NaHCO_3_. The alternation of the surface of (CuGa)_0.5_ZnS_2_ might cause deactivation of sacrificial CO_2_ reduction, as shown in Figure 5. Next, DRS of a mixture of (CuGa)_0.5_ZnS_2_ and RGO-(CoO_*x*_/BiVO_4_) of a Z-scheme photocatalyst was investigated. The Z-scheme photocatalyst before CO_2_ reduction had a risen baseline (Figure 6(d)) because of RGO (Figure 6(g)).^24^ However, the different risen baseline from the RGO was also observed after the Z-schematic CO_2_ reduction (Figure 6(e), (f)), especially using 100 mmol L^–1^ NaHCO_3_. The shape of the risen baseline observed after Z-schematic CO_2_ reduction (Figure 6(e), (f)) was similar to that after sacrificial CO_2_ reduction (Figure 6(b), (c)). Moreover, the surface composition of Ga, Zn, and S to Cu also decreased after the Z-schematic CO_2_ reduction (Table S2). (CuGa)_0.5_ZnS_2_ also changed during Z-schematic CO_2_ reduction, especially using 100 mmol L^–1^ NaHCO_3_.

**Figure 7:**
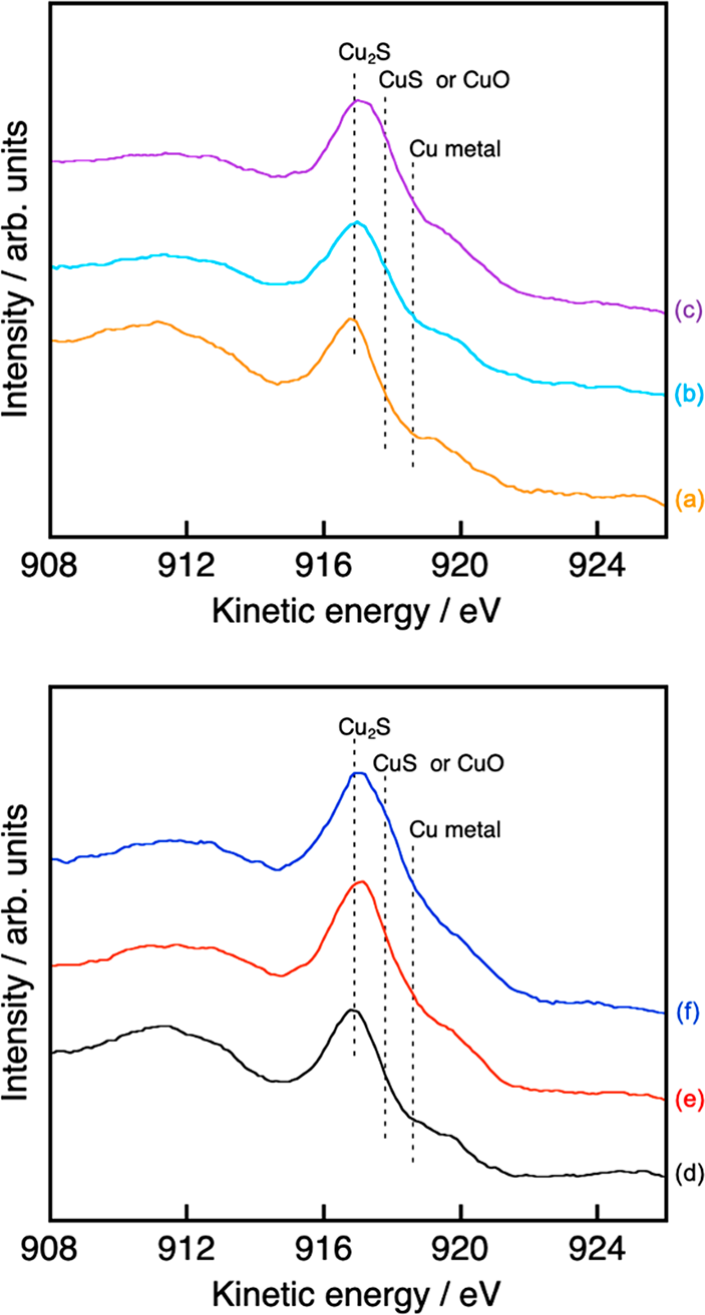
Auger spectra for Cu L_3_M_4,5_M_4,5_ of (a) (CuGa)_0.5_ZnS_2_, (CuGa)_0.5_ZnS_2_ after sacrificial CO_2_ reduction (b) without NaHCO_3_ and (c) with 1.0 mol L^–1^ NaHCO_3_ , and a Z-scheme photocatalyst of a mixture of (CuGa)_0.5_ZnS_2_ and RGO-(CoO_*x*_/BiVO_4_) (d) before CO_2_ reduction and after CO_2_ reduction (e) using 10 mmol L^–1^ NaHCO_3_ and (f) using 100 mmol L^–1^ NaHCO_3_. The kinetic energy was corrected using a C 1s peak for (a)–(c) and an In 3d peak for (d)–(f).

**Figure 8:**
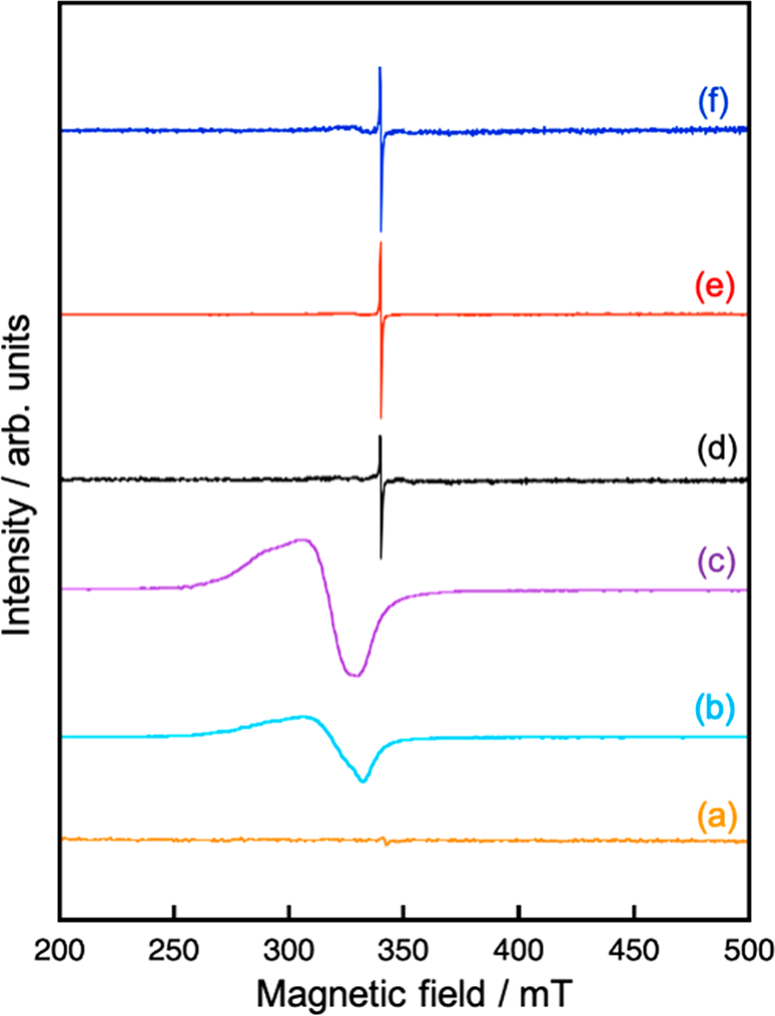
ESR at 77 K of (a) (CuGa)_0.5_ZnS_2_, (CuGa)_0.5_ZnS_2_ after sacrificial CO_2_ reduction (b) without NaHCO_3_ and (c) with 1.0 mol L^–1^ NaHCO_3_, and a Z-scheme photocatalyst of a mixture of (CuGa)_0.5_ZnS_2_ and RGO-(CoO_*x*_/BiVO_4_) (d) before CO_2_ reduction and after CO_2_ reduction (e) using 10 mmol L^–1^ NaHCO_3_ and (f) using 100 mmol L^–1^ NaHCO_3_. The intensity of (b) and (c) is 1/5.

The chemical state of Cu should also affect the durability of photocatalytic CO_2_ reduction. An Auger peak of Cu L_3_M_4,5_M_4,5_ of (CuGa)_0.5_ZnS_2_ before and after sacrificial CO_2_ reduction was measured using an XPS instrument attached with a Mg target to reveal the chemical state of Cu on the surface of (CuGa)_0.5_ZnS_2_ as shown in Figure 7. ESR was also measured at 77 K to clarify the chemical state of Cu in the bulk in addition to the surface of (CuGa)_0.5_ZnS_2_, as shown in Figure 8, because Cu^2+^ is an ESR-active species but Cu^+^ is not. First, we determined whether the chemical state of Cu in (CuGa)_0.5_ZnS_2_ changes before and after the sacrificial CO_2_ reduction. The (CuGa)_0.5_ZnS_2_ before sacrificial CO_2_ reduction gave a sharp peak around 917 eV of kinetic energy (Figure 7(a)), agreeing with reported Cu^+^ at 916.7–917.3 eV for Cu_2_S.^27,28^ This is reasonable because Cu^+^ in (CuGa)_0.5_ZnS_2_ should be the major species. On the other hand, the slight peak shift to a high kinetic energy was observed, and the shoulder peak around 917.8–918.0 eV corresponding to Cu^2+^ of CuS and CuO^28,29^ appeared in the (CuGa)_0.5_ZnS_2_ after sacrificial CO_2_ reduction (Figure 7(b), (c)), in which (CuGa)_0.5_ZnS_2_ after sacrificial CO_2_ reduction using NaHCO_3_ showed a larger peak for the oxidized Cu than that without NaHCO_3_. An obvious signal of Cu^0^ around 918.6–918.8 eV was not observed. No ESR signals were observed for (CuGa)_0.5_ZnS_2_ before the CO_2_ reduction (Figure 8(a)), whereas an obvious signal appeared after the sacrificial CO_2_ reduction (Figure 8(b), (c)). The *g* value of 2.1 was close to the reported values (2.0–2.4) of Cu^2+^.^30,31^ It was also confirmed using Cu(1 wt %)-loaded ZnS treated with H_2_ reduction that the ESR signal was not due to Cu^0^. These AES and ESR measurements revealed a part of Cu^+^ of (CuGa)_0.5_ZnS_2_ was oxidized to Cu^2+^ during the CO_2_ reduction even in the presence of a sacrificial electron donor. Thus, the deactivation observed in sacrificial CO_2_ reduction in Figure 5 was also due to Cu^2+^ formation, which could work as a recombination site or kill an active site. The huge self-oxidation to form Cu^2+^ might be due to adsorption of some ions as discussed above. Next, AES and ESR for a Z-scheme photocatalyst are compared before and after CO_2_ reduction, as shown in Figure 2(b), (c). An ESR signal of RGO (Figure S4) was observed for an RGO-(CoO_*x*_/BiVO_4_) composite. However, obvious ESR signals due to Cu^2+^ were not observed for the Z-scheme photocatalysts before and after Z-schematic CO_2_ reduction (Figure 8(d)–(f)), being different from that after sacrificial CO_2_ reduction (Figure 8(b), (c)). We also confirmed that the mixture of (CuGa)_0.5_ZnS_2_ after the sacrificial CO_2_ reduction and RGO-(CoO_*x*_/BiVO_4_) gave the signal of Cu^2+^ as a blank test. In contrast to the ESR, peak shifts and obvious shoulder peaks of Cu^2+^ were observed in the AES for the Z-scheme photocatalyst after CO_2_ reduction (Figure 7(e), (f)), being similar to that after sacrificial CO_2_ reduction, although the signal of Cu^2+^ was not clear in that before Z-schematic CO_2_ reduction (Figure 7(d)). The shoulder peak assigned to CuS or CuO in the Z-scheme photocatalyst after CO_2_ reduction using 100 mmol L^–1^ NaHCO_3_ was larger than that using 10 mmol L^–1^ NaHCO_3_, while the peak of the Z-scheme photocatalyst after CO_2_ reduction using 10 mmol L^–1^ NaHCO_3_ also shifted to high kinetic energy. The difference in the intensity for the shoulder peak might suggest that the Z-scheme photocatalyst after CO_2_ reduction using 100 mmol L^–1^ NaHCO_3_ contained more Cu^2+^ than that using 10 mmol L^–1^ NaHCO_3_. Although a part of Cu^+^ on the surface of (CuGa)_0.5_ZnS_2_ was slightly photooxidized to Cu^2+^ during the Z-schematic CO_2_ reduction, Cu^+^ in the bulk was not, indicating Cu^2+^ was suppressed in Z-schematic CO_2_ reduction compared with that in sacrificial CO_2_ reduction. Therefore, Z-schematic CO_2_ reduction proceeded more stably than sacrificial CO_2_ reduction, as shown in Figures 2 and 5.

### Photoelectrochemical CO_2_ Reduction Using a (CuGa)_0.5_ZnS_2_ Photocathode under Visible Light Irradiation

**Figure 9:**
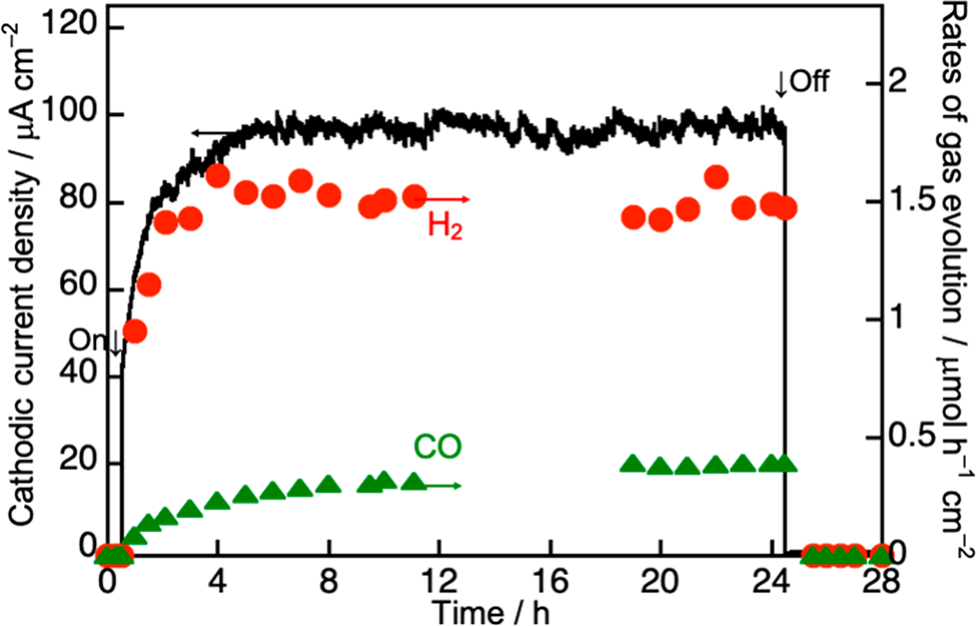
Photoelectrochemical CO_2_ reduction under visible light irradiation over a (CuGa)_0.5_ZnS_2_ photocathode in an aqueous solution containing 0.1 mol L^–1^ KHCO_3_ at a constant applied bias of 0.1 V vs RHE. Photoelectrode: drop cast, electrolyte: 0.1 mol L^–1^ KHCO_3(aq)_ with dissolved CO_2_ at 1 atm, light source: 300 W Xe lamp (λ > 420 nm), applied bias: 0.1 V vs RHE (−0.5 V vs Ag/AgCl (pH 6.9)), reference electrode: Ag/AgCl, counter electrode: Pt.

Photoelectrochemical measurement is beneficial to reduce CO_2_. Moreover, the photoelectrochemical property of the employed photocatalyst material is also important to consider in a Z-scheme photocatalyst system using RGO, because the electron flow mechanism is similar between the Z-scheme system with RGO and the photoelectrode system with an electric wire.^12,32^ In other words, the condition to give a high photoelectrochemical performance should be preferable for an efficient Z-scheme system. Photoelectrochemical CO_2_ reduction was examined using a (CuGa)_0.5_ZnS_2_ photocathode under visible light irradiation. A (CuGa)_0.5_ZnS_2_ powder/FTO electrode gave a cathodic photocurrent by irradiating visible light under both Ar and CO_2_ (Figure S5). The photocurrent and the onset potential under CO_2_ were similar to those under Ar. We also evaluated the photoelectrochemical property under acidic and neutral pH in the presence of CO_2_ (Figure S6). The photocurrent density around pH 4 was much smaller than that around neutral pH, indicating the presence of a basic salt was also effective for the photoelectrochemical property for bare (CuGa)_0.5_ZnS_2_. In addition, efficient performance of the present Z-scheme system by tuning the pH higher than pH 4 is reasonable judging from the photoelectrochemical measurement. Since the similarity (Figure S5) does not mean CO_2_ reduction, bulk electrolysis was conducted to determine the Faradaic efficiency. Photoelectrochemical CO_2_ reduction over the (CuGa)_0.5_ZnS_2_ photocathode using an aqueous KHCO_3_ solution under visible light irradiation at −0.5 V vs Ag/AgCl at pH 6.9 (0.1 V vs RHE) was carried out as shown in Figure 9. The cathodic current was not observed under dark conditions, whereas (CuGa)_0.5_ZnS_2_ gave a steady cathodic current by irradiating visible light after a 5 h induction period. The reduction products of H_2_ and CO were obtained with 79% and 21% Faradaic efficiencies, respectively, giving almost 100% total Faradaic efficiency. The Faradaic efficiencies of H_2_ and CO formation were almost constant after the induction period. These results indicated that (CuGa)_0.5_ZnS_2_ could reduce CO_2_ to CO without obvious deactivation in a photoelectrochemical cell under visible light irradiation, being different from Z-schematic CO_2_ reduction in which slight deactivation was observed. The photoelectrochemical CO_2_ reduction with high stability was due to efficient hole transportation from the metal sulfide to the FTO substrate by applying a suitable potential. Thus, we successfully constructed a photoelectrochemical CO_2_ reduction system using a metal sulfide photocathode based on powdered materials working under visible light irradiation. The photoelectrochemical CO_2_ reduction system has an advantage from the viewpoint of collection of evolved syngas without separating it from evolved O_2_.

## Conclusions

The (CuGa)_0.5_ZnS_2_ photocatalyst reduced CO_2_ to CO using SO_3_^2–^ as a sacrificial electron donor under visible light irradiation. Adding NaHCO_3_ into a reactant solution of the sacrificial CO_2_ reduction enhanced the CO evolution and gave 42% of the selectivity for CO formation even without any cocatalysts. A Z-scheme photocatalyst consisting of the bare (CuGa)_0.5_ZnS_2_ and RGO-(CoO_*x*_/BiVO_4_) reduced CO_2_ to CO using water as an electron donor without any salt additives under visible light irradiation. Moreover, adding a basic salt into the reactant solution improved CO evolution, reaching 10–20% of the CO selectivity accompanied by almost stoichiometric O_2_ evolution. The enhancement of Z-schematic CO_2_ reduction was due to the suitable pH condition and efficient supply of hydrated CO_2_ molecules of a reactant by adding a basic salt. The basic additive with suitable concentration also stabilized the Z-scheme photocatalyst using a photocorrosive metal sulfide material in CO_2_ reduction. Thus, reactant solution tuning contributed to both improvement and stabilization of the Z-schematic CO_2_ reduction. In addition to the reactant solution control, we successfully enhanced the Z-schematic CO_2_ reduction by employing an improved (CuGa)_0.5_ZnS_2_ photocatalyst. The present Z-scheme system was also active for CO_2_ reduction to CO using water as an electron donor under simulated sunlight. The Z-schematic CO_2_ reduction proceeded more stably than the sacrificial CO_2_ reduction, because self-photooxidation of Cu^+^ on (CuGa)_0.5_ZnS_2_ in the Z-schematic CO_2_ reduction was suppressed compared with that in the sacrificial CO_2_ reduction judging from the AES and ESR measurements. Photoelectrochemical CO_2_ reduction was also demonstrated using our original powdered (CuGa)_0.5_ZnS_2_ photocatalyst. Usually, PEC systems are composed of high-quality thin films prepared by a complex process and require surface modification with some thin-layer compounds and a cocatalyst. In contrast, the present PEC using (CuGa)_0.5_ZnS_2_ gave a reasonable efficiency even when employing simple powdered materials and without such a surface modification. The Faradaic efficiency for CO formation reached 21% at 0.1 V vs RHE with high stability because of efficient hole transportation to the FTO substrate. Thus, a PEC cell with a (CuGa)_0.5_ZnS_2_ photocathode was beneficial to demonstrate efficient and stable CO_2_ reduction. Moreover, it indicates that the (CuGa)_0.5_ZnS_2_ photocatalyst itself possesses an excellent electrocatalytic site for CO_2_ reduction on the surface. Thus, photocatalytic CO_2_ reduction with high activity, selectivity, and durability using water as an electron donor was achieved under visible light irradiation by employing a metal sulfide photocatalyst in a simple aqueous suspension and photoelectrochemical systems. Our finding about tuning the reactant solution will contribute to the construction of efficient Z-scheme and photoelectrochemical systems employing metal sulfide photocatalysts for CO_2_ reduction using water as an electron donor, namely, accompanied by O_2_ evolution, under visible light irradiation. We expect that highly selective CO_2_ reduction will further be achieved by introducing a suitable active site and surface modification of the metal sulfide photocatalyst for Z-schematic and photoelectrochemical CO_2_ reduction.

## Supporting Information Available

Additional characterization, Z-schematic reaction, and photoelectrochemical measurement (PDF)
