# Supplementary material for: Photocatalytic CO2 Reduction Using Water as an Electron Donor under Visible Light Irradiation by Z-Scheme and Photoelectrochemical Systems over (CuGa)0.5ZnS2 in the Presence of Basic Additives
Source: J Am Chem Soc. 2022 Jan 25;144(5):2323–32. doi: 10.1021/jacs.1c12636 (PMC8832390; doi:10.1021/jacs.1c12636)
Supplement: Supplementary file 1 — ja1c12636_si_001.pdf [file ja1c12636_si_001.pdf]

Supporting Information

Photocatalytic CO_2 Reduction Using Water as an Electron Donor under Visible Light Irradiation by Z-Scheme and Photoelectrochemical Systems over $(CuGa)_{0.5}ZnS_2$ in the Presence of Basic Additives

Shunya Yoshino,[a] Akihide Iwase,[a] Yuichi Yamaguchi,[a] Tomiko M. Suzuki,[b] Takeshi Morikawa[b] and Akihiko Kudo[a*]

[a]*Department of Applied Chemistry, Faculty of Science, Tokyo University of Science, 1-3 Kagurazaka, Shinjuku-ku, Tokyo 162-8601, Japan*

[b]*Toyota Central R & D Labs., Inc., 41-1 Yokomichi, Nagakute, Aichi 480-1192, Japan*

Email: a-kudo@rs.tus.ac.jp

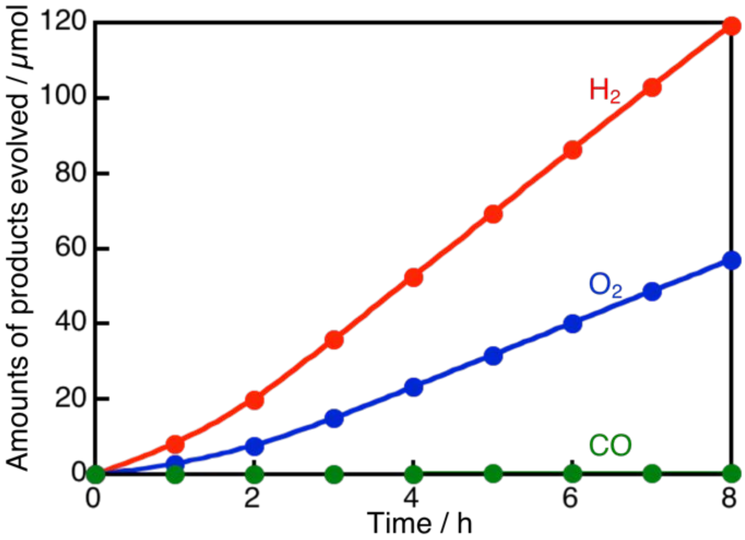

Figure S1. Z-schematic water splitting under visible light irradiation using bare-$(CuGa)_{0.5}ZnS_2$ and RGO-(CoO_x/$BiVO_4$) photocatalysts. Photocatalyst: 0.05 g each, reactant solution: water without any additives (120 mL, pH6.3), flow gas: Ar (1 atm), light source : 300 W Xe lamp ($\lambda > 420$ nm), light irradiation area: 33 cm^2, cell: top-irradiation cell with a Pyrex window.

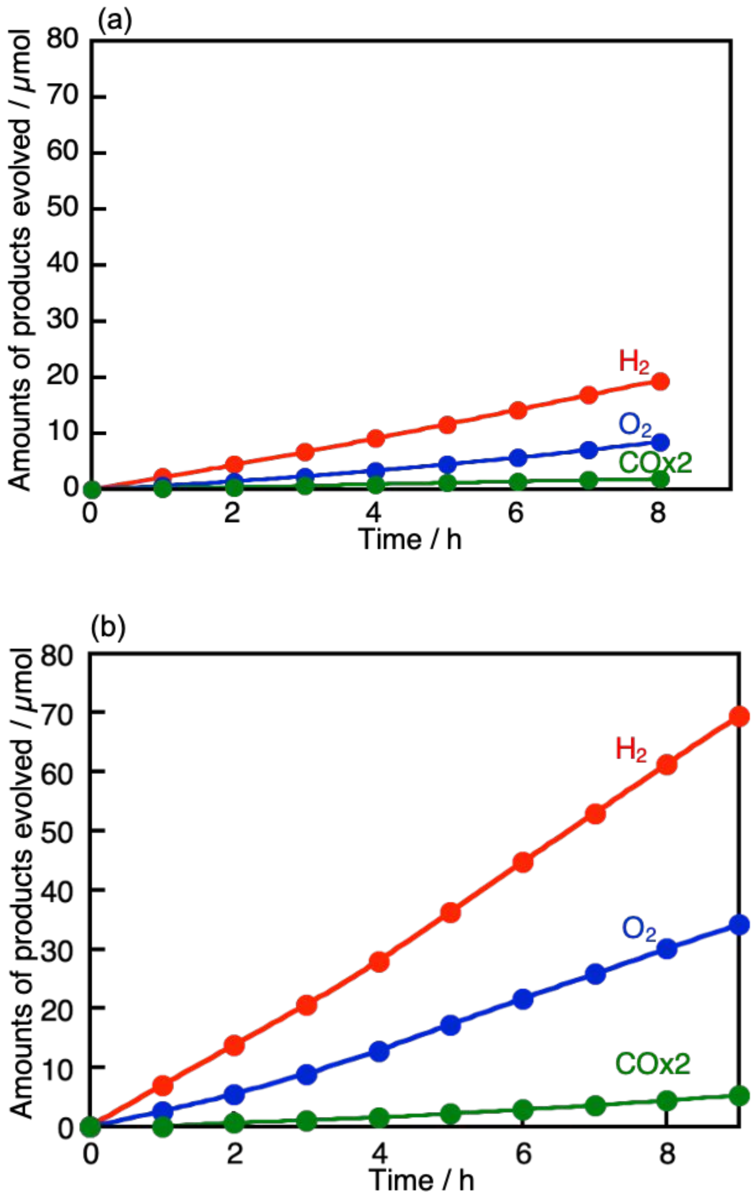

Figure S2. Z-schematic CO_2 reduction under visible light irradiation using (a) $CuGaS_2$ or (b) $(CuGa)_{0.5}ZnS_2$ prepared by a SSR and RGO-($CoO_x/BiVO_4$) photocatalysts in the presence of 10 mmol L^{-1} $NaHCO_3$. Photocatalyst: 0.05 g each, reactant solution: 10 mmol L^{-1} $NaHCO_3$ (120 mL), flow gas: CO_2 (1 atm), light source : 300 W Xe lamp (λ > 420 nm), light irradiation area: 33 cm^2, cell: top-irradiation cell with a Pyrex window.

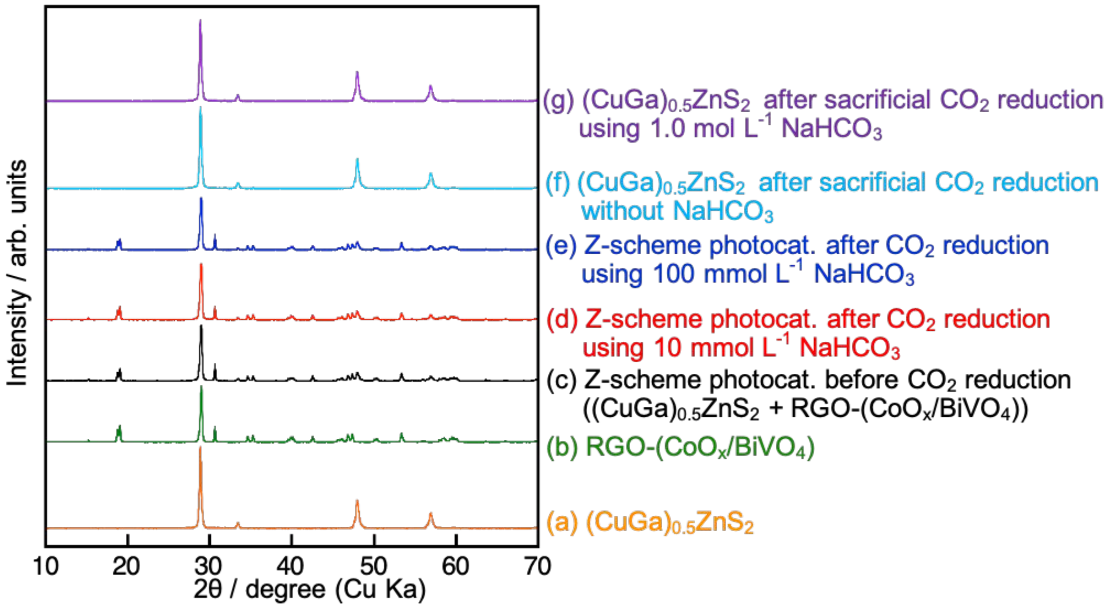

Figure S3. XRD of (a) $(CuGa)_{0.5}ZnS_2$, (b) RGO-($CoO_x/BiVO_4$), (c) a Z-scheme photocatalyst of mixture of $(CuGa)_{0.5}ZnS_2$ and RGO-($CoO_x/BiVO_4$) before CO_2 reduction, (d) a Z-scheme photocatalyst after CO_2 reduction using 10 mmol L^{-1} $NaHCO_3$, (e) a Z-scheme photocatalyst after CO_2 reduction using 100 mmol L^{-1} $NaHCO_3$, (f) $(CuGa)_{0.5}ZnS_2$ after sacrificial CO_2 reduction without $NaHCO_3$, and (g) $(CuGa)_{0.5}ZnS_2$ after sacrificial CO_2 reduction using 1.0 mol L^{-1} $NaHCO_3$.

Table S1 Ratio of XPS peak areas for $(CuGa)_{0.5}ZnS_2$ before and after sacrificial CO_2 reduction using $NaHCO_3$

$(CuGa)_{0.5}ZnS_2$ before or after sacrificial CO_2 reduction	Reactant solution	Ratio of peak areas		
		$Ga3d/Cu2p_{3/2}$	$Zn2p_{3/2}/Cu2p_{3/2}$	$S2p/Cu2p_{3/2}$
Before	-	0.22	1.90	1.81
After	0.1 mol L^{-1} $K_2SO_{3\ (aq.)}$	0.10	0.83	0.89
After	0.1 mol L^{-1} K_2SO_3 +1.0 mol L^{-1} $NaHCO_{3\ (aq.)}$	0.08	0.57	0.75

Table S2 Ratio of XPS peak areas for Z-scheme photocatalysts consisting of $(CuGa)_{0.5}ZnS_2$ and RGO-($CoO_x/BiVO_4$) before and after Z-schematic CO_2 reduction using $NaHCO_3$

Z-scheme photocatalyst before or after CO_2 reduction	Reactant solution	Ratio of peak areas		
		$Ga3p/Cu2p_{3/2}$	$Zn2p_{3/2}/Cu2p_{3/2}$	$S2s/Cu2p_{3/2}$
Before	-	0.24	2.35	0.36
After	10 mmol L^{-1} $NaHCO_{3\ (aq.)}$	0.09	0.66	0.17
After	100 mmol L^{-1} $NaHCO_{3\ (aq.)}$	0.07	0.58	0.14

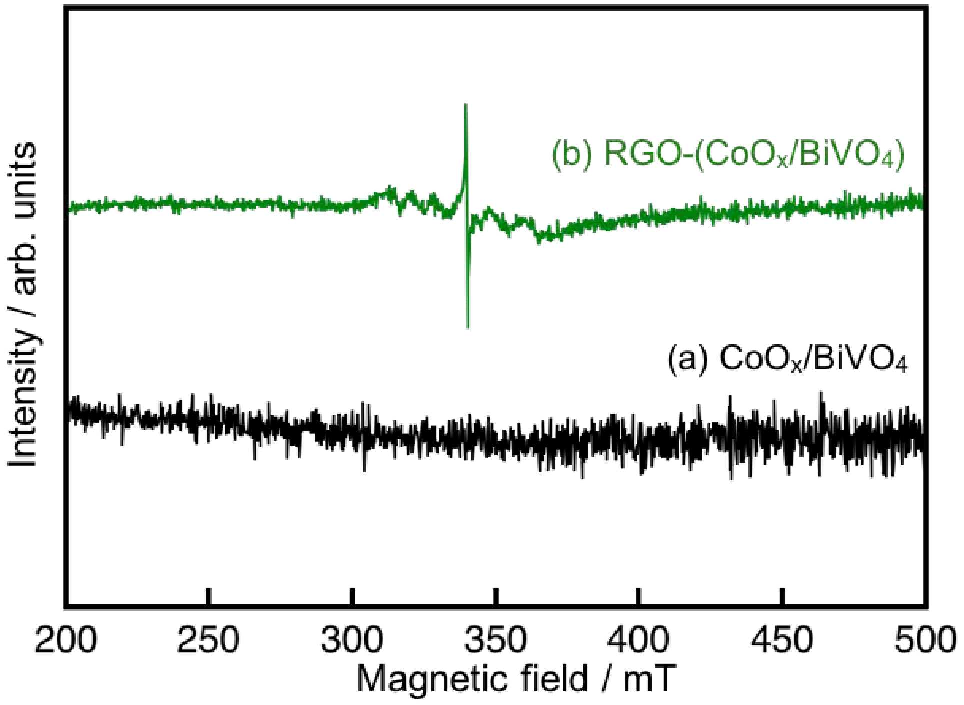

Figure S4. ESR at 77 K of (a) $CoO_x/BiVO_4$ and (b) RGO-($CoO_x/BiVO_4$).

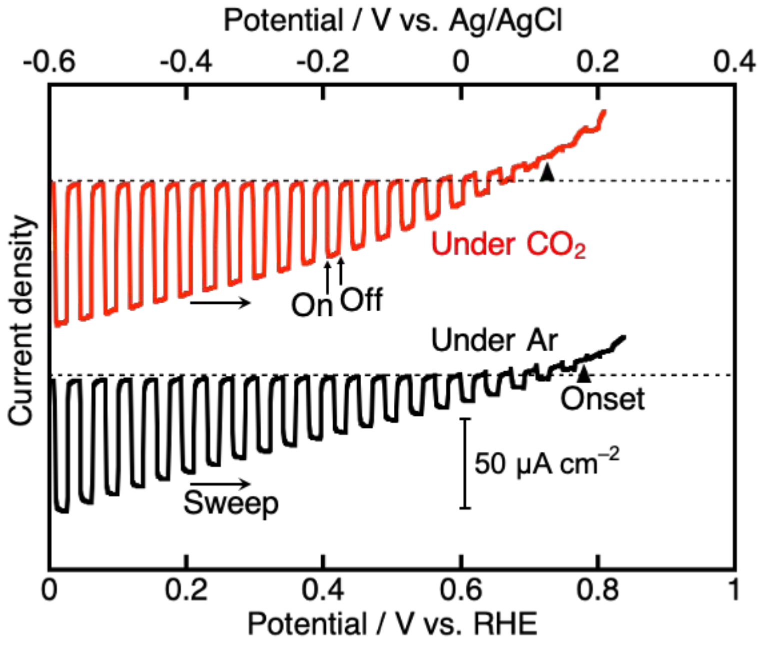

Figure S5. Current vs. potential curves of a $(CuGa)_{0.5}ZnS_2$ photocathode under CO_2 and Ar irradiated with visible light. Electrolyte: 0.1 mol L^{-1} $KHCO_3$ under 1 atm CO_2 (pH 6.9) or 0.1 mol L^{-1} K_2SO_4 with phosphate buffer under 1 atm Ar (pH 6.9).

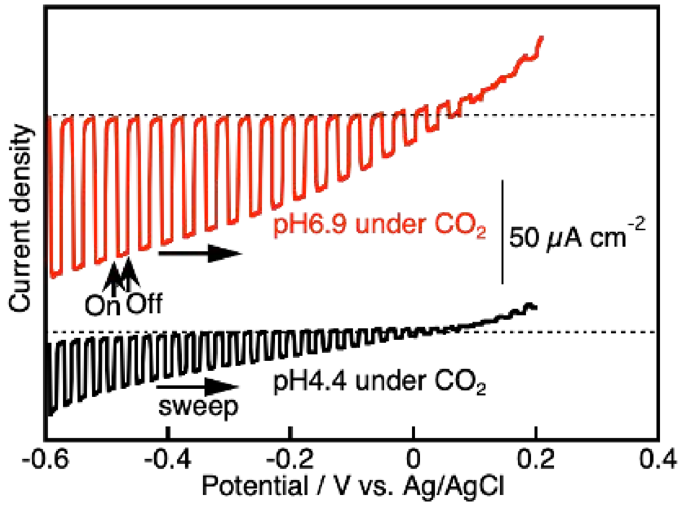

Figure S6. Current vs. potential curves of a $(CuGa)_{0.5}ZnS_2$ photocathode under CO_2 at pH4.4 and 6.9 irradiated with visible light. Electrolyte: 0.1 mol L^{-1} $KHCO_3$ $_{(aq.)}$ (pH 6.9) or 0.1 mol L^{-1} K_2SO_4 $_{(aq.)}$ (pH 4.4) under 1 atm CO_2.
